# New-onset atrial fibrillation and associated outcomes and resource use among critically ill adults—a multicenter retrospective cohort study

**DOI:** 10.1186/s13054-020-2730-0

**Published:** 2020-01-13

**Authors:** Shannon M. Fernando, Rebecca Mathew, Benjamin Hibbert, Bram Rochwerg, Laveena Munshi, Allan J. Walkey, Morten Hylander Møller, Trevor Simard, Pietro Di Santo, F. Daniel Ramirez, Peter Tanuseputro, Kwadwo Kyeremanteng

**Affiliations:** 1 Division of Critical Care, Department of Medicine, University of Ottawa, Ottawa, ON Canada; 2 Department of Emergency Medicine, University of Ottawa, Ottawa, ON Canada; 3 Division of Cardiology, University of Ottawa Heart Institute, Ottawa, ON Canada; 4 Department of Medicine, Division of Critical Care, McMaster University, Hamilton, ON Canada; 5 Department of Health Research Methods, Evidence, and Impact, McMaster University, Hamilton, ON Canada; 6 Interdepartmental Division of Critical Care Medicine, University of Toronto, Toronto, ON Canada; 7 Department of Medicine, Sinai Health System, Toronto, ON Canada; 8 Department of Medicine, The Pulmonary Center, Boston University School of Medicine, Boston, MA USA; 9 Center for Implementation and Improvement Sciences, Boston University School of Medicine, Boston, MA USA; 10 Department of Intensive Care, Copenhagen University Hospital Righospitalet, Copenhagen, Denmark; 11 Electrophysiology Service, Hôpital Cardiologique du Haut-Lévêque, Centre Hospitalier Universitaire de Bordeaux, Bordeaux-Pessac, France; 12 L’Institut de Rythmologie et Modélisation Cardiaque, Université de Bordeaux, Bordeaux-Pessac, France; 13 Clinical Epidemiology Program, Ottawa Hospital Research Institute, Ottawa, ON Canada; 14 Bruyere Research Institute, Ottawa, ON Canada; 15 Division of Palliative Care, Department of Medicine, University of Ottawa, Ottawa, ON Canada; 16 School of Epidemiology and Public Health, University of Ottawa, Ottawa, ON Canada; 17 Institut du Savoir Montfort, Ottawa, ON Canada

**Keywords:** Atrial fibrillation, Critical care, Intensive care unit, Resource utilization, Costs

## Abstract

**Background:**

New-onset atrial fibrillation (NOAF) is commonly encountered in critically ill adults. Evidence evaluating the association between NOAF and patient-important outcomes in this population is conflicting. Furthermore, little is known regarding the association between NOAF and resource use or hospital costs.

**Methods:**

Retrospective analysis (2011–2016) of a prospectively collected registry from two Canadian hospitals of consecutive ICU patients aged ≥ 18 years. We excluded patients with a known history of AF prior to hospital admission. Any occurrence of atrial fibrillation (AF) was prospectively recorded by bedside nurses. The primary outcome was hospital mortality, and we used multivariable logistic regression to adjust for confounders. We used a generalized linear model to evaluate contributors to total cost.

**Results:**

We included 15,014 patients, and 1541 (10.3%) had NOAF during their ICU admission. While NOAF was not associated with increased odds of hospital death among the entire cohort (adjusted odds ratio [aOR] 1.02 [95% confidence interval [CI] 0.97–1.08]), an interaction was noted between NOAF and sepsis, and the presence of both was associated with higher odds of hospital mortality (aOR 1.28 [95% CI 1.09–1.36]) than either alone. Patients with NOAF had higher total costs (cost ratio [CR] 1.09 [95% CI 1.02–1.20]). Among patients with NOAF, treatment with a rhythm-control strategy was associated with higher costs (CR 1.24 [95% CI 1.07–1.40]).

**Conclusions:**

While NOAF was not associated with death or requiring discharge to long-term care among critically ill patients, it was associated with increased length of stay in ICU and increased total costs.

## Introduction

Atrial fibrillation (AF) is the most common cardiac dysrhythmia, with a lifetime risk of 1 in 4 among older adults [1]. Development of AF has been associated with stroke, myocardial infarction, heart failure, and death [2]. In the intensive care unit (ICU), patients often present with pre-existing AF; however, some ICU patients may develop new-onset AF (NOAF) in the context of critical illness [3]. Unlike AF seen in non-critically ill patients, NOAF is often thought to be a consequence of critical illness pathophysiology and treatment, including inflammation, electrolyte disturbances, or proarrhythmic medications, namely vasopressors and inotropes [4]. Incidence of NOAF in the general ICU varies markedly; however, most studies suggest that 10–15% of patients will develop this complication during their ICU stay [3–5].

The clinical importance of critical illness-associated NOAF is a matter of ongoing uncertainty [4]. AF in and of itself may contribute to clinical decompensation through hemodynamic compromise [6]. Alternatively, NOAF may simply represent a marker of increased illness severity, and may identify patients at increased risk of death without acting causally to worsen prognosis. Some cohort studies have identified an independent association between NOAF and mortality [7–9], while others have not [5, 10]. Whether this relationship is seen among all critically ill patients, or limited to select subgroups, is unknown. Furthermore, the factors associated with death among patients with NOAF are unknown.

Equally important is the relationship between NOAF, and subsequent ICU resource use and costs [11, 12]. Since the ICU is a major source of hospital expenditure, considerable effort has been dedicated to understand the contributors to cost, in order to drive policy and optimize resource utilization [13]. With regard to NOAF, little is known regarding the degree of impact on cost expenditures. We primarily sought to evaluate the association between NOAF and outcomes, resource utilization, and costs among critically ill adult patients. Given the prognostic importance of NOAF among patients with sepsis [14, 15], we secondarily aimed to evaluate the association between incidence of NOAF and associated outcomes and resource utilization among critically ill patients with suspected infection, sepsis, and septic shock.

## Materials and methods

We obtained ethics approval for this study from the Ottawa Health Science Network Research Ethics Board (protocol 20160570-01H).

### Study design, setting, and subjects

We studied ICU patients from two hospitals within the Ottawa Hospital network (Ottawa, ON). These hospitals have approximately 2500 combined ICU admissions per year. These are combined medical and non-cardiac surgical ICUs. We retrospectively examined prospectively collected data from the Ottawa Hospital Data Warehouse, a health administrative database used in previous studies [16–19]. From hospital admission, data is gathered daily from each patient and stored in the Data Warehouse. Data quality assessments are executed routinely, and quality-assurance initiatives are conducted regularly to ensure completeness and accuracy.

We included all consecutive patients ≥ 18 years of age, admitted to one of the two ICUs between January 2011 and December 2016. Sample size was determined pragmatically, on the basis of available patients in the Data Warehouse. We also examined pre-specified subgroups of patients, including those with suspected infection, sepsis, and septic shock, as based on the Third International Consensus Definitions for Sepsis and Septic Shock (Sepsis-3) [20–22]. “Suspected infection” was defined as concomitant administration of oral or parenteral antibiotics, and sampling of body-fluid cultures, as performed previously [16, 23], and in keeping with the Sepsis-3 definitions [20]. “Sepsis” was defined as suspected infection and an increase in the Sequential Organ Failure Assessment (SOFA) score by greater than 2 points [20, 21]. Finally, “septic shock” was defined by sepsis in addition to initiation of vasopressors or a serum lactate ≥ 2.0 mmol/L [20, 22].

### Data collection

We obtained all data from the Ottawa Hospital Data Warehouse. We abstracted demographic data, comorbidities, Elixhauser Comorbidity Score [24], and Multiple Organ Dysfunction Score (MODS) [25] at the time of ICU admission. The Elixhauser Comorbidity Score is generated from comorbidities stored in the Data Warehouse, and the association between this index and hospital mortality has been previously validated in our database [26]. The “most responsible diagnosis” was recorded at death or discharge, based upon International Classification of Diseases, Version 10 (ICD-10, July 2015). We also noted whether there was presence of a “No cardiopulmonary resuscitation (CPR)” directive at the time of ICU admission. We collected outcome data from admission until either the point of discharge from hospital or hospital death.

We determined patient costs using the case-costing system of the Ottawa Hospital Data Warehouse, as done previously [17, 23, 27]. Total hospital costs include both direct and indirect sources. Direct costs refer to all hospital expenses with fee codes linked to the patient identifier. Indirect costs refer to any overhead operational fees associated with provided service. The Ottawa Hospital uses a standardized case-costing methodology, developed by the Ontario Case Costing Initiative, and based upon the Canadian Institute for Health Information Management guidelines [28]. Costs were indexed to 2018 Canadian Dollars using consumer price indices [23, 27].

### Outcomes

The primary outcome was hospital mortality. Secondary outcomes included discharge directly from hospital to long-term care (among survivors to hospital discharge originally from home), hospital readmission within 30 days of hospital discharge among survivors, ICU length of stay (LOS), hospital LOS, resource utilization (including invasive and non-invasive mechanical ventilation, and renal replacement therapy), and total hospital costs.

### Screening for atrial fibrillation

For each patient, the occurrence of any AF was prospectively recorded by bedside nurses for the purposes of quality assurance. The date and time of AF, as captured by the bedside nurse, was stored in the Data Warehouse. Patients identified through this method were then evaluated by a single investigator (SMF), to confirm the diagnosis. Since there is no consensus definition for NOAF [4], we followed pre-existing definitions from the literature [7, 8]. NOAF was defined as either (1) AF ≥ 1 h in duration, as noted by bedside telemetry (routinely evaluated in charts where electrocardiograms were not completed); (2) AF < 1 h in duration, but captured on electrocardiogram; or (3) AF initiating pharmacologic therapy or electrical cardioversion. All bedside ECGs, along with final interpretation by an attending cardiologist are stored in patient records. “Sustained” AF was defined as failure to convert to sinus rhythm 24 h following the onset of any pharmacological treatment or electrical cardioversion. We excluded patients with a previously documented or known history of AF, as determined at the time of hospital admission and stored in the Data Warehouse.

### Statistical analysis

We performed all statistical analyses using R (version 3.3.3) and IBM SPSS (version 24.0). We present data as mean values, with standard deviation (SD), or medians, with interquartile range (IQR), where appropriate. Student’s *t* test (parametric values), Mann-Whitney test (non-parametric values), and *χ*^2^ (for categorical values) were performed to determine between-group differences. In keeping with existing guidelines, we did not perform pairwise comparisons of baseline characteristics [29]. To adjust for measured confounders in the association between new-onset AF and outcomes of interest, we followed recommendations for observational studies in the critically ill [30]. As per these recommendations, confounders were determined a priori, on the basis of their likelihood of influencing both the presence of NOAF and mortality and not acting as mediators or colliders in the association between AF and mortality, as based upon existing clinical knowledge evaluating the association between NOAF and mortality in critically ill patients [3, 4]. In accordance with these recommendations [30], we used multivariable logistic regression modeling to adjust for important continuous (age, MODS at ICU admission, Elixhauser comorbidity index) and categorical (sex, individual comorbidities, “No-CPR” directive on admission, location prior to ICU admission, and most responsible diagnosis) variables. As recommended, variables on the causal pathway and potentially contributing to NOAF (e.g., vasoactive medications) were not included [30]. We evaluated for possible synergy between NOAF and sepsis through the use of an interaction term in the primary model, as performed previously [23]. If a statistically significant interaction term was found between NOAF and sepsis, we then represented this with a four-level categorization. We assessed variation in total hospital costs using a multivariable generalized linear model with gamma distribution and log link [31, 32]. We present adjusted odds ratios (aORs) and cost ratios (CRs) with 95% confidence intervals. A *P* value of ≤ 0.05 was considered statistically significant.

## Results

**Fig. 1:**
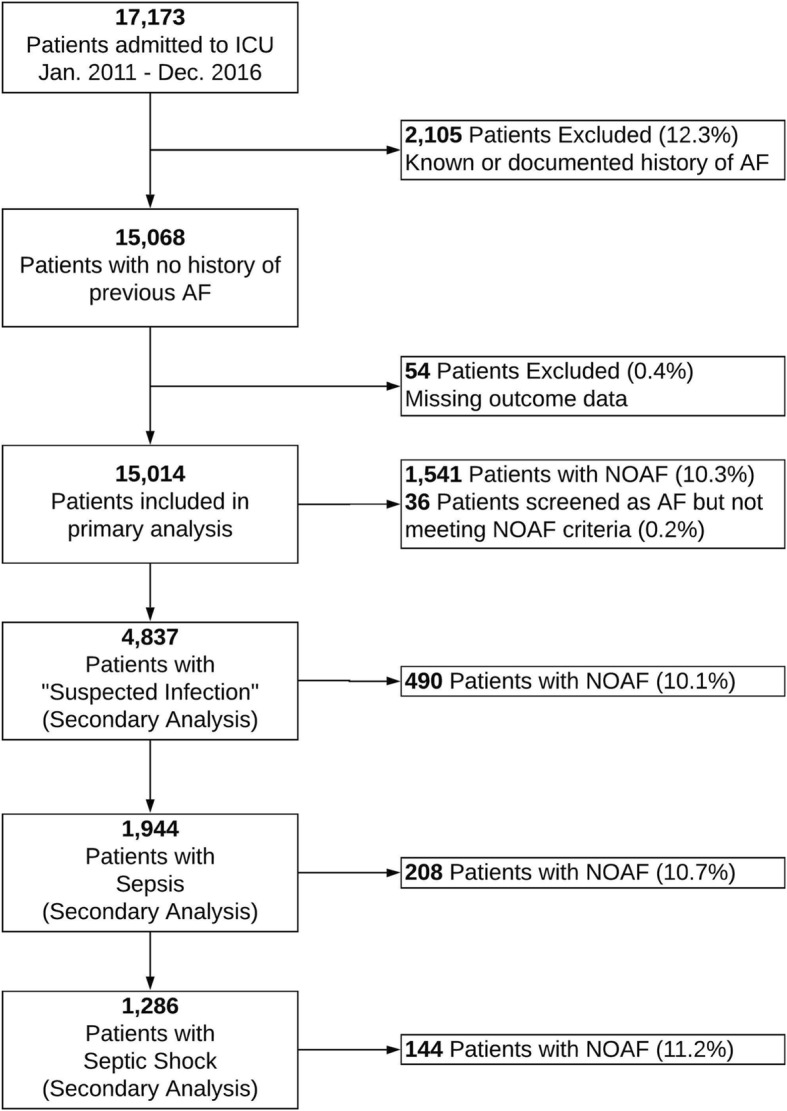
Study flow diagram. AF, atrial fibrillation; ICU, intensive care unit; NOAF, new-onset atrial fibrillation

A total of 17,173 patients were admitted to the participating ICUs from 2011 to 2016 (Fig. 1). Of these, 2105 patients (12.3%) had a known or documented history of AF prior to ICU admission, and were excluded. A further 54 patients (0.4%) were excluded because of missing outcome data.

We included 15,014 patients in the analyses. Of these patients, 1541 (10.3%) had NOAF while in the ICU. Baseline characteristics of patient with and without NOAF are shown in Table 1. NOAF patients were older (mean age 64.7 years vs. 58.5 years), had higher severity of illness (mean MODS 5.3 vs. 4.2), and higher comorbidity burden.

**Table 1:** Characteristics of ICU patients (*n* = 15,014) with and without new-onset atrial fibrillation (NOAF)

Characteristic	New-onset atrial fibrillation (*n* = 1541)	No atrial fibrillation (*n* = 13,473)
Age, years, mean (SD)	64.7 (17.6)	58.7 (12.2)
Male, *n* (%)	854 (55.4)	7114 (52.8)
ICU MODS, mean (SD)	5.3 (2.4)	4.2 (2.7)
Comorbidities		
Congestive heart failure	223 (14.5)	869 (6.4)
Peripheral vascular disease	124 (8.0)	418 (3.1)
Hypertension	525 (34.1)	3341 (24.8)
Chronic obstructive pulmonary disease	176 (11.4)	1414 (10.5)
Diabetes mellitus	427 (27.7)	3422 (25.4)
Chronic kidney disease	129 (8.4)	1037 (7.7)
Liver disease	63 (4.1)	528 (3.9)
Malignancy	221 (14.3)	1874 (13.9)
Alcohol misuse	81 (5.3)	732 (5.4)
Psychosis	23 (1.5)	139 (1.0)
Depression	41 (2.7)	288 (2.1)
Elixhauser Comorbidity Score, mean (SD)	5.3 (4.1)	4.1 (3.7)
Daytime ICU admission (0800–1700), *n* (%)	932 (60.5)	8472 (62.9)
Location prior to ICU admission, *n* (%)		
Emergency department	653 (42.4)	5930 (44.0)
Hospital wards	501 (32.5)	4374 (32.5)
Operating room	78 (5.1)	754 (5.6)
Peripheral hospital	309 (20.1)	2415 (17.9)
Previous ED visits, mean (SD)^a^	2.3 (2.5)	2.3 (2.2)
Previous ICU admissions, mean (SD)^a^	0.3 (1.0)	0.7 (1.1)
Previous ICU days, mean (SD)^a^	2.3 (5.4)	2.0 (5.0)
No CPR directive at admission, *n* (%)	299 (19.4)	1480 (11.0)
Most responsible diagnosis, *n* (%)		
Infection/sepsis	349 (22.6)	2037 (15.1)
Respiratory failure	289 (18.8)	2732 (20.3)
Trauma	137 (8.9)	1388 (10.3)
Malignancy	117 (7.6)	1283 (9.5)
Spontaneous intracranial hemorrhage	99 (6.4)	1182 (8.8)
Stroke	90 (5.8)	1073 (8.0)
Overdose/poisoning	36 (2.3)	432 (3.2)
Renal failure	41 (2.6)	399 (3.0)
Gastrointestinal bleeding	57 (3.7)	321 (2.4)
Congestive heart failure	104 (6.7)	409 (3.0)
Cardiac arrest	22 (1.4)	188 (1.4)
Seizures/status epilepticus	18 (1.2)	202 (1.5)
Diabetic ketoacidosis	9 (0.6)	116 (0.9)
Other	173 (11.2)	1711 (12.7)

*Abbreviations: CPR* cardiopulmonary resuscitation, *ED* emergency department, *ICU* intensive care unit, *MODS* Multiple Organ Dysfunction Score, *SD* standard deviation

^a^Only including patients with previous visits to the Ottawa Hospital

Patient outcomes are depicted in Table 2. Median time from hospitalization to development of NOAF was 1 day (IQR 1–3), and 345 (22.4%) of NOAF patients had sustained AF lasting longer than 24 h. Multivariable logistic regression analyses examining in-hospital mortality among the entire cohort, and among subgroups with suspected infection (*n =* 4837, Table 3), sepsis (*n* = 1944, 40.2% of “suspected infection” population), and septic shock (*n* = 1286, 66.2% of “sepsis” population) are included in Additional files 1, 2, and 3: Tables S1-S3, respectively). Following adjustment for confounding variables, NOAF was not associated with higher hospital mortality among all ICU patients (aOR 1.02 [95% CI 0.97–1.08]). However, NOAF was associated with higher hospital mortality among ICU patients with suspected infection (aOR 1.21 [95% CI 1.08–1.37]), sepsis (aOR 1.24 [95% CI 1.10–1.39]), and septic shock (aOR 1.28 [95% CI 1.14–1.44]). A statistically significant interaction was seen between NOAF and presence of sepsis, and the presence of both was associated with higher odds than either alone (Additional file 4: Table S4). No difference was seen with regard to disposition to home among survivors (aOR 0.95 [95% CI 0.88–1.09]), readmission to ICU during hospitalization (aOR 1.04 [95% CI 0.95–1.11]), or hospital readmission within 30 days of discharge (aOR 1.08 [95% CI 0.87–1.19]). Patients with NOAF had prolonged median ICU LOS (7 days vs. 6 days, *P* < 0.001) and median total hospital LOS (14 days vs. 12 days, *P* < 0.001). Among patients with NOAF, factors associated with increased risk of hospital mortality included increasing age, increased MODS score, history of CHF (as identified in the Data Warehouse), and sustained AF (Additional file 5: Table S5).

**Table 2:** Outcomes of ICU patients (*n* = 15,014) with and without new-onset atrial fibrillation (NOAF)

Characteristic	New-onset atrial fibrillation (*n* = 1541)	No atrial fibrillation (*n* = 13,473)	Adjusted odds ratio^c^ (95% CI)	*P* value
In-hospital mortality, *n* (%)	576 (37.4)	4034 (29.9)	1.02 (0.97–1.08)	0.31
Disposition, *n* (%)^a^			0.95 (0.88–1.09)	0.57
Home	562 (58.2)	6230 (66.0)		
Long-term care center	403 (41.8)	3209 (34.0)		
Time to NOAF development from hospital admission, days, median (IQR)	1 (1–3)	–		
Persistent atrial fibrillation, *n* (%)^b^	345 (22.4)	–		
ICU length of stay, days, median (IQR)	7 (4–14)	6 (2–9)		< 0.001
Hospital length of stay, days, median (IQR)	14 (8–29)	12 (4–25)		< 0.001
Ventilator-free days, median (IQR)	6 (3–8)	6 (4–10)		0.03
Readmission to ICU during hospitalization, *n* (%)	225 (14.6)	1670 (12.4)	1.04 (0.94–1.11)	0.27
Readmission within 30 days from discharge, *n* (%)^a^	294 (30.5)	2426 (25.7)	1.08 (0.87–1.19)	0.13

*Abbreviations: ICU* intensive care unit, *IQR* interquartile range, *MODS* Multiple Organ Dysfunction Score, *SD* standard deviation

^a^Only includes patients surviving to discharge (*n* = 10,404)

^b^Defined as presence of any atrial fibrillation following 24 h of treatment

^c^Ratio of NOAF to patients with no atrial fibrillation

**Table 3:** Multivariable logistic regression model for hospital mortality for patients with suspected infection (*n* = 4837)

Variable	Odds ratio	95% CI	*P* value
Age (per 5 years)	1.06	1.03–1.11	< 0.001
Male gender	1.04	0.93–1.14	0.41
New-onset atrial fibrillation	1.21	1.08–1.37	< 0.001
MODS (per 1 point)	1.07	1.04–1.10	< 0.001
Comorbidities			
Congestive heart failure	1.28	1.04–1.55	< 0.01
Peripheral vascular disease	1.05	0.83–1.20	0.65
Hypertension	0.97	0.85–1.08	0.48
Chronic obstructive pulmonary disease	1.06	1.03–1.09	< 0.01
Diabetes mellitus	1.04	0.90–1.17	0.22
Chronic kidney disease	1.08	0.97–1.19	0.10
Liver disease	1.14	1.06–1.23	< 0.01
Alcohol misuse	0.97	0.88–1.15	0.63
Elixhauser Comorbidity Score (per 1 point)	1.02	1.01–1.04	< 0.01
No CPR directive at ICU admission	1.64	1.32–2.01	< 0.001
Location prior to ICU admission			
Hospital wards	Ref		
Emergency department	1.16	0.91–1.30	0.42
Operating room	1.10	0.92–1.21	0.31
Peripheral hospital	0.96	0.84–1.13	0.58

*Abbreviations: MODS* Multiple Organ Dysfunction Score, *ICU* intensive care unit, *CI* confidence interval, *CPR* cardiopulmonary resuscitation

Comparisons of resources used between patients with and without AF are shown in Table 4. No differences were seen in the use of invasive (54.7% in those with NOAF vs. 52.8% in those without NOAF, *P* = 0.16) or non-invasive ventilation (16.6% in those with NOAF vs. 17.5% in those without NOAF, *P* = 0.38). Vasoactive medication use was higher among patients with NOAF (64.3% vs. 61.2%, *P* = 0.02). In terms of treatment strategy for NOAF, 747 (48.5%) patients received antiarrhythmic medical therapy (i.e., amiodarone, procainamide, or flecainide), while 644 (41.8%) received therapy with a beta-blocker, calcium channel blocker, or digoxin. A total of 128 patients (8.3%) received a combination of the above therapies.

**Table 4:** Resource utilization among ICU patients (*n* = 15,014) with and without new-onset atrial fibrillation (NOAF)

Characteristic	New-onset atrial fibrillation (*n* = 1541)	No atrial fibrillation (*n* = 13,473)	*P* value
Invasive mechanical ventilation, *n* (%)	843 (54.7)	7119 (52.8)	0.16
Invasive mechanical ventilation days, median (IQR)	5 (3–6)	5 (2–7)	0.37
Non-invasive mechanical ventilation, *n* (%)	256 (16.6)	2358 (17.5)	0.38
Non-invasive mechanical ventilation days, median (IQR)	1 (1–2)	1 (1–2)	0.15
Vasoactive medication, *n* (%)	991 (64.3)	8245 (61.2)	0.02
Vasoactive medication days, median (IQR)	5 (3–9)	5 (2–10)	0.11
Arterial line, *n* (%)	915 (59.4)	8407 (62.4)	0.02
Arterial line days, median (IQR)	5 (4–10)	5 (4–9)	0.36
Renal replacement therapy, *n* (%)	304 (19.7)	2411 (17.9)	0.18
Renal replacement therapy days, median (IQR)	4 (2–9)	4 (2–9)	0.57
NEMS/day, mean (SD)	26.3 (7.2)	25.1 (8.0)	0.35
Transfusion products, *n* (%)			
Packed red blood cells	592 (38.4)	5079 (37.7)	0.58
Platelets	104 (6.7)	688 (5.1)	< 0.01
Fresh frozen plasma	85 (5.5)	835 (6.2)	0.29
Albumin	735 (47.7)	6898 (51.2)	< 0.01
Treatment strategy, *n* (%)			
Beta-blocker, calcium channel blocker, or digoxin	644 (41.8)	–	
Antiarrhythmic	747 (48.5)	–	
Electrical cardioversion	22 (1.4)	–	
Combination	128 (8.3)	–	

*Abbreviations: IQR* interquartile range, *NEMS* Nine Equivalents of Nursing Manpower Scale, *SD* standard deviation

Finally, comparisons of patient costs between patients with and without NOAF are shown in Table 5. Mean total costs were higher among NOAF patients ($50,799 vs. $41,832, *P* < 0.001), as were mean total ICU costs ($41,303 vs. $28,298, *P* < 0.01). Total cost per survivor for patients with NOAF was $81,120, as compared to $59,710 for patients without NOAF. Presence of NOAF in our ICU cohort was a significant predictor of total hospital costs (CR 1.09 [95% CI 1.02–1.21], Additional file 6: Table S6). Among patients with NOAF (Additional file 7: Table S7), significant predictors of total hospital costs include total hospital or ICU LOS, use of invasive mechanical ventilation or renal replacement therapy, and use of antiarrhythmic medical therapy (as compared to beta-blocker, calcium channel blocker, or digoxin treatment).

**Table 5:** Costs among ICU patients (*n* = 15,014) with and without new-onset atrial fibrillation (NOAF)

Characteristic	New-onset atrial fibrillation (*n* = 1541)	No atrial fibrillation (*n* = 13,473)	*P* value
Total costs, $, mean (SD)	50,799 (38,473)	41,832 (38,772)	< 0.001
Total direct costs, $, mean (SD)	37,392 (31,538)	30,231 (32,922)	< 0.01
Total cost per survivor, $	81,120	59,710	
Attributable costs, $, mean (SD)			
Food services	1046 (1104)	937 (1032)	0.37
Health professionals (non-physician or nursing)	2859 (2175)	2957 (2.250)	0.45
Laboratory	2144 (1910)	1877 (1823)	< 0.01
Medical imaging	1438 (1672)	1401 (1573)	0.29
Pharmacy	3582 (3288)	2893 (3092)	< 0.001
Respiratory therapy	3944 (6188)	3748 (6229)	0.48
Nursing	39,492 (36,493)	28,344 (31,302)	< 0.001
ICU costs, $, mean (SD)			
Total costs	41,303 (37,743)	28,298 (41,393)	< 0.001
Direct costs	31,874 (30,838)	21,323 (21,504)	< 0.001
Indirect costs	9239 (8482)	7367 (8043)	0.08

*Abbreviations: IQR* interquartile range, *NEMS* Nine Equivalents of Nursing Manpower Scale, *SD* standard deviation

## Discussion

We identified NOAF in 10.3% of critically ill adults, in keeping with known prevalence rates [4]. We found no association between NOAF and increased hospital mortality in critically ill adults, although an association was demonstrated in subgroups of patients with suspected infection, sepsis, and septic shock. Among patients who developed NOAF, predictors of hospital mortality included increasing age, severity of illness, history of CHF, and sustained AF following treatment. Patients with NOAF had prolonged ICU and hospital LOS, and NOAF was a predictor of increased total costs. Mechanical ventilation, renal replacement therapy, and use of antiarrhythmic therapy were significant predictors of total cost in NOAF patients. Taken together, our study identifies important novel associations between NOAF and outcomes among critically ill patients, and also describes the economic impact of NOAF.

Hospital mortality among critically ill adults is high, and therefore, identification of prognostic factors associated with increased risk can be helpful to clinicians in escalating or tailoring therapy. Identification of these factors may also be helpful in discussions with patients and families regarding goals of care. NOAF is often thought to be a marker of illness severity [3]; however, the evidence examining the association between NOAF and hospital mortality remains uncertain [4]. In our large cohort of critically ill adults, we did not find such an association. However, evaluation of subgroups of patients with suspected infection, sepsis, and septic shock did find an independent association between NOAF and hospital mortality potentially suggesting that the consequences of NOAF in the patient with sepsis may differ from other populations.

Important physiologic changes occur during sepsis that make the atrial substrate vulnerable to NOAF [33], and patients with sepsis have a nearly sixfold risk of developing AF, as compared to other populations [14]. Our findings identifying an independent association between NOAF and mortality from sepsis are supportive of existing literature, and potentially suggest that survival in this population may be improved through the prevention and treatment of NOAF [34]. Importantly, unlike previous studies, we defined sepsis and septic shock using the most recent Sepsis-3 definitions, indicating that NOAF has potential implications in these populations, and demonstrate an interaction between the presence of NOAF and sepsis. In keeping with the Sepsis-3 focus of sepsis as infection with concomitant organ dysfunction, NOAF may indeed represent sepsis-defining cardiac dysfunction [35]. As such, NOAF during sepsis might mediate mortality and may not simply be a marker of illness severity, as is seen in other disease processes [36].

If NOAF does represent a marker of illness severity, then its presence may be considered an important indicator of deterioration among critically ill patients. Therefore, identification of factors associated with mortality among ICU patients who develop NOAF remains an important area of ongoing research [4]. Unsurprisingly, a history of heart failure was associated with hospital mortality among patients with NOAF in our cohort, suggesting that these patients may be more susceptible to the hemodynamic effects of NOAF. Our results also found that sustained AF following 24 h of treatment was also associated with higher hospital mortality. Therefore, sustained AF may represent a particular ominous marker of illness severity among critically ill adults, and clinicians should act cautiously in those patients with NOAF and persisting AF.

We additionally evaluated the association between NOAF and hospital resource use and cost. Identification of factors associated with such outcomes remains an important focus in critical care research [11]. Few differences were found with regard to resource utilization between patients with and without NOAF. However, patients with NOAF did have prolonged ICU and hospital LOS, which translated into higher costs. The presence of NOAF was also a significant predictor of costs among our cohort, and this was independent of other important factors more commonly associated with cost, including renal replacement therapy and mechanical ventilation [37]. This prolonged LOS was also manifested in increased laboratory, pharmacy, and nursing costs. Despite higher costs, patients with NOAF had higher unadjusted mortality, which translated into significant differences in cost per survivor (a proxy indicator of cost-effectiveness).

Among patients with NOAF, treatment with antiarrhythmic medication (as compared to beta-blockers, calcium channel blockers, or digoxin) was associated with higher cost, but decrease in mortality. While this study was not designed to test the efficacy of therapeutic agents for NOAF, the little evidence that exists on this topic seems to suggest no beneficial effect of any particular agent [3, 4]. This is in keeping with our results. We did not find evidence of difference in mortality associated with antiarrhythmic agents, but did note increased cost. This is likely attributable to the expense of these drugs [38], and the fact that they often cannot be administered outside of a monitored setting and therefore may prolong ICU LOS. However, it must be stressed that the efficacy of these agents can only be appropriately tested in a randomized trial, and while equipoise exists, decisions related to agents for treatment of NOAF must be made on a case-by-case basis.

We used a large multicenter database of patients with prospective identification of NOAF, and provide novel data related to hospital mortality, resource utilization, and cost. We also closely followed recommendations for control of confounding in observational studies [30]. However, our study has important limitations. Most importantly, our database lacks the granularity to investigate other potential factors that may influence outcome from NOAF, such as underlying cardiac function, pulmonary artery catheterization, rate of AF, timing of AF (particularly in relation to onset of critical illness), use of anticoagulation, and incidence of other arrhythmias. Some outcome data, such as incidence of stroke, were not available. We additionally excluded patients with existing AF on the basis of known or documented AF, but it is possible that patients may have had pre-existing AF and not known, due to absence of symptoms [39]. Overall, this lack of granularity represents an important limitation in our study. Second, patients were included on the basis of prospective identification by nursing staff. While similar methods have been used at other institutions [40], it is possible that cases of NOAF were missed using this methodology, particularly if AF was brief and transiently resolved. Third, we only had data related to outcomes prior to hospital death or discharge. Existing data suggest deleterious long-term outcomes in patients who develop NOAF during critical illness [15, 41]. Unfortunately, we were unable to evaluate this in our cohort. Finally, while our data are derived from two hospitals, they exist within the same city and therefore are susceptible to bias from local practices.

## Conclusions

While NOAF was not associated with hospital mortality among all critically ill patients, it was associated with mortality in subgroups of patients with suspected infection, sepsis, and septic shock. Among patients with NOAF, sustained AF was associated with higher risk of hospital mortality. Finally, patients with NOAF had higher costs than patients without NOAF, and NOAF was a predictor of increased total costs among all ICU patients.

## Supplementary information

**Additional file 1 : Table S1.** Multivariable Logistic Regression Model for hospital mortality for entire study cohort (*n* = 15,014). Multivariable Logistic Regression Model for hospital mortality for entire study cohort (*n* = 15,014).

**Additional file 2 : Table S2.** Multivariable Logistic Regression Model for hospital mortality for patients with sepsis (*n* = 1944). Multivariable Logistic Regression Model for hospital mortality for patients with sepsis (*n* = 1944).

**Additional file 3 : Table S3.** Multivariable Logistic Regression Model for hospital mortality for patients with septic shock (*n* = 1286). Multivariable Logistic Regression Model for hospital mortality for patients with septic shock (*n* = 1286).

**Additional file 4 : Table S4.** Multivariable Logistic Regression Model for hospital mortality for entire study cohort (*n* = 15,014), including interaction terms. Multivariable Logistic Regression Model for hospital mortality for entire study cohort (*n* = 15,014), including interaction terms.

**Additional file 5 : Table S5.** Multivariable Logistic Regression Model for hospital mortality among patients with new-onset atrial fibrillation (*n* = 1541). Multivariable Logistic Regression Model for hospital mortality among patients with new-onset atrial fibrillation (*n* = 1541).

**Additional file 6 : Table S6.** Generalized Linear Model with gamma distribution and log link for total cost for entire study cohort (*n* = 15,014). Generalized Linear Model with gamma distribution and log link for total cost for entire study cohort (*n* = 15,014).

**Additional file 7 : Table S7.** Generalized Linear Model with gamma distribution and log link for total cost for patients with new-onset atrial fibrillation (*n* = 15,014). Generalized Linear Model with gamma distribution and log link for total cost for patients with new-onset atrial fibrillation (*n* = 15,014).

## Data Availability

The datasets generated and analyzed are not publicly available due to patient privacy considerations, but are available from the corresponding author on reasonable request.

## Abbreviations

AF: Atrial fibrillation
aOR: Adjusted odds ratio
CPR: Cardiopulmonary resuscitation
CR: Cost ratio
ICD-10: International Classifications of Diseases, Version 10
ICU: Intensive care unit
IQR: Interquartile range
LOS: Length of stay
MODS: Multiple Organ Dysfunction Score
NOAF: New-onset atrial fibrillation
SD: Standard deviation
Sepsis-3: Third International Consensus Definitions for Sepsis and Septic Shock
SOFA: Sequential Organ Failure Assessment
