# Supplementary material for: New-onset atrial fibrillation and associated outcomes and resource use among critically ill adults—a multicenter retrospective cohort study
Source: Crit Care. 2020 Jan 13;24:15. doi: 10.1186/s13054-020-2730-0 (PMC6958729; doi:10.1186/s13054-020-2730-0)
Supplement: Supplementary file 1 — Additional file 1 : Table S1. Multivariable Logistic Regression Model for hospital mortality for entire study cohort (n = 15,014). Multivariable Logistic Regression Model for hospital mortality for entire study cohort (n = 15,014). [file 13054_2020_2730_MOESM1_ESM.docx]

**Table S1**: Multivariable Logistic Regression Model for hospital mortality for entire study cohort (*n* = 15,014). *Abbreviations:* MODS = Multiple Organ Dysfunction Score; ICU = Intensive Care Unit; CI = confidence interval; CPR = cardiopulmonary resuscitation

| **Variable** | **Odds Ratio** | **95% CI** | ***P Value*** |
| --- | --- | --- | --- |
| **Age (per 5 years)** | 1.04 | 1.02-1.09 | <0.001 |
| **Male Gender** | 0.96 | 0.88-1.09 | 0.26 |
| **New-Onset Atrial Fibrillation** | 1.02 | 0.97-1.08 | 0.18 |
| **MODS (per 1 point)** | 1.10 | 1.08-1.13 | <0.001 |
| **Comorbidities** |  |  |  |
| Congestive Heart Failure | 1.43 | 1.12-1.75 | <0.001 |
| Peripheral Vascular Disease | 1.12 | 0.87-1.34 | 0.32 |
| Hypertension | 0.94 | 0.83-1.06 | 0.44 |
| Chronic Obstructive Pulmonary Disease | 1.09 | 1.01-1.18 | <0.01 |
| Diabetes Mellitus | 1.05 | 0.92-1.16 | 0.18 |
| Chronic Kidney Disease | 1.06 | 0.94-1.20 | 0.14 |
| Liver Disease | 1.12 | 1.02-1.23 | <0.01 |
| Alcohol Misuse | 0.94 | 0.80-1.15 | 0.34 |
| **Elixhauser Comorbidity Score (per 1 point)** | 1.03 | 1.01-1.06 | 0.02 |
| **No CPR Directive at ICU Admission** | 1.77 | 1.41-2.03 | <0.001 |
| **Location Prior to ICU Admission** |  |  |  |
| Hospital Wards | Ref |  |  |
| Emergency Department | 1.12 | 0.89-1.23 | 0.52 |
| Operating Room | 1.15 | 0.96-1.28 | 0.08 |
| Peripheral Hospital | 0.95 | 0.82-1.17 | 0.43 |
| **Most Responsible Diagnosis** |  |  |  |
| Other | Ref |  |  |
| Infection/Sepsis | 0.89 | 0.76-1.08 | 0.41 |
| Respiratory Failure | 1.36 | 1.15-1.52 | <0.001 |
| Trauma | 0.91 | 0.76-1.10 | 0.14 |
| Malignancy | 1.03 | 0.86-1.25 | 0.93 |
| Spontaneous Intracranial Hemorrhage | 1.88 | 1.44-2.33 | <0.001 |
| Stroke | 1.52 | 1.17-1.96 | <0.001 |
| Overdose/Poisoning | 0.47 | 0.33-0.68 | <0.001 |
| Renal Failure | 0.38 | 0.24-0.59 | <0.001 |
| Gastrointestinal Bleeding | 1.15 | 0.77-1.71 | 0.55 |
| Congestive Heart Failure | 1.06 | 0.68-1.69 | 0.94 |
| Cardiac Arrest | 1.74 | 1.42-2.07 | <0.001 |
| Seizures/Status Epilepticus | 0.65 | 0.47-1.02 | 0.06 |
| Diabetic Ketoacidosis | 0.63 | 0.35-1.03 | 0.06 |
