# Supplementary material for: New-onset atrial fibrillation and associated outcomes and resource use among critically ill adults—a multicenter retrospective cohort study
Source: Crit Care. 2020 Jan 13;24:15. doi: 10.1186/s13054-020-2730-0 (PMC6958729; doi:10.1186/s13054-020-2730-0)
Supplement: Supplementary file 2 — Additional file 2 : Table S2. Multivariable Logistic Regression Model for hospital mortality for patients with sepsis (n = 1944). Multivariable Logistic Regression Model for hospital mortality for patients with sepsis (n = 1944). [file 13054_2020_2730_MOESM2_ESM.docx]

**Table S2**: Multivariable Logistic Regression Model for hospital mortality for patients with sepsis (*n* = 1,944). *Abbreviations:* MODS = Multiple Organ Dysfunction Score; ICU = Intensive Care Unit; CI = confidence interval; CPR = cardiopulmonary resuscitation

| **Variable** | **Odds Ratio** | **95% CI** | ***P Value*** |
| --- | --- | --- | --- |
| **Age (per 5 years)** | 1.05 | 1.04-1.15 | <0.001 |
| **Male Gender** | 1.03 | 0.91-1.13 | 0.48 |
| **New-Onset Atrial Fibrillation** | 1.24 | 1.10-1.39 | <0.001 |
| **MODS (per 1 point)** | 1.04 | 1.03-1.07 | <0.001 |
| **Comorbidities** |  |  |  |
| Congestive Heart Failure | 1.30 | 1.06-1.55 | <0.01 |
| Peripheral Vascular Disease | 1.04 | 0.84-1.18 | 0.72 |
| Hypertension | 0.98 | 0.86-1.10 | 0.45 |
| Chronic Obstructive Pulmonary Disease | 1.05 | 1.02-1.07 | <0.01 |
| Diabetes Mellitus | 1.03 | 0.88-1.15 | 0.35 |
| Chronic Kidney Disease | 1.10 | 0.99-1.20 | 0.05 |
| Liver Disease | 1.15 | 1.07-1.25 | <0.001 |
| Alcohol Misuse | 0.99 | 0.90-1.12 | 0.77 |
| **Elixhauser Comorbidity Score (per 1 point)** | 1.02 | 1.01-1.03 | <0.01 |
| **No CPR Directive at ICU Admission** | 1.71 | 1.38-2.16 | <0.001 |
| **Location Prior to ICU Admission** |  |  |  |
| Hospital Wards | Ref |  |  |
| Emergency Department | 1.14 | 0.88-1.28 | 0.51 |
| Operating Room | 1.12 | 0.94-1.24 | 0.27 |
| Peripheral Hospital | 0.98 | 0.86-1.15 | 0.55 |
