# Supplementary material for: New-onset atrial fibrillation and associated outcomes and resource use among critically ill adults—a multicenter retrospective cohort study
Source: Crit Care. 2020 Jan 13;24:15. doi: 10.1186/s13054-020-2730-0 (PMC6958729; doi:10.1186/s13054-020-2730-0)
Supplement: Supplementary file 3 — Additional file 3 : Table S3. Multivariable Logistic Regression Model for hospital mortality for patients with septic shock (n = 1286). Multivariable Logistic Regression Model for hospital mortality for patients with septic shock (n = 1286). [file 13054_2020_2730_MOESM3_ESM.docx]

**Table S3**: Multivariable Logistic Regression Model for hospital mortality for patients with septic shock (*n* = 1,286). *Abbreviations:* MODS = Multiple Organ Dysfunction Score; ICU = Intensive Care Unit; CI = confidence interval; CPR = cardiopulmonary resuscitation

| **Variable** | **Odds Ratio** | **95% CI** | ***P Value*** |
| --- | --- | --- | --- |
| **Age (per 5 years)** | 1.06 | 1.04-1.13 | <0.001 |
| **Male Gender** | 1.02 | 0.90-1.14 | 0.53 |
| **New-Onset Atrial Fibrillation** | 1.28 | 1.14-1.44 | <0.001 |
| **MODS (per 1 point)** | 1.05 | 1.04-1.07 | <0.001 |
| **Comorbidities** |  |  |  |
| Congestive Heart Failure | 1.32 | 1.08-1.58 | <0.001 |
| Peripheral Vascular Disease | 1.05 | 0.87-1.19 | 0.66 |
| Hypertension | 1.01 | 0.88-1.14 | 0.42 |
| Chronic Obstructive Pulmonary Disease | 1.06 | 1.03-1.09 | <0.01 |
| Diabetes Mellitus | 1.02 | 0.89-1.13 | 0.39 |
| Chronic Kidney Disease | 1.14 | 1.01-1.22 | 0.03 |
| Liver Disease | 1.18 | 1.08-1.25 | <0.001 |
| Alcohol Misuse | 0.97 | 0.89-1.10 | 0.73 |
| **Elixhauser Comorbidity Score (per 1 point)** | 1.02 | 1.01-1.03 | <0.01 |
| **No CPR Directive at ICU Admission** | 1.76 | 1.41-2.20 | <0.001 |
| **Location Prior to ICU Admission** |  |  |  |
| Hospital Wards | Ref |  |  |
| Emergency Department | 1.13 | 0.89-1.30 | 0.46 |
| Operating Room | 1.10 | 0.92-1.22 | 0.31 |
| Peripheral Hospital | 0.96 | 0.88-1.12 | 0.50 |
