# Supplementary material for: New-onset atrial fibrillation and associated outcomes and resource use among critically ill adults—a multicenter retrospective cohort study
Source: Crit Care. 2020 Jan 13;24:15. doi: 10.1186/s13054-020-2730-0 (PMC6958729; doi:10.1186/s13054-020-2730-0)
Supplement: Supplementary file 4 — Additional file 4 : Table S4. Multivariable Logistic Regression Model for hospital mortality for entire study cohort (n = 15,014), including interaction terms. Multivariable Logistic Regression Model for hospital mortality for entire study cohort (n = 15,014), including interaction terms. [file 13054_2020_2730_MOESM4_ESM.docx]

**Table S4**: Multivariable Logistic Regression Model for hospital mortality for entire study cohort (*n* = 15,014), including interaction terms. *Abbreviations:* NOAF = New-onset Atrial Fibrillation; MODS = Multiple Organ Dysfunction Score; ICU = Intensive Care Unit; CI = confidence interval; CPR = cardiopulmonary resuscitation

| **Variable** | **Odds Ratio** | **95% CI** | ***P Value*** |
| --- | --- | --- | --- |
| **Age (per 5 years)** | 1.03 | 1.01-1.10 | <0.01 |
| **Male Gender** | 0.97 | 0.87-1.09 | 0.31 |
| **NOAF-Sepsis Interaction** |  |  |  |
| NOAF-; Sepsis- | Ref |  |  |
| NOAF-; Sepsis+ | 1.14 | 1.03-1.25 | <0.01 |
| NOAF+; Sepsis- | 0.96 | 0.91-1.03 | 0.12 |
| NOAF+; Sepsis+ | 1.28 | 1.09-1.36 | <0.001 |
| **MODS (per 1 point)** | 1.10 | 1.08-1.13 | <0.001 |
| **Comorbidities** |  |  |  |
| Congestive Heart Failure | 1.41 | 1.08-1.77 | <0.001 |
| Peripheral Vascular Disease | 1.10 | 0.85-1.36 | 0.39 |
| Hypertension | 0.96 | 0.85-1.10 | 0.47 |
| Chronic Obstructive Pulmonary Disease | 1.07 | 0.99-1.15 | 0.07 |
| Diabetes Mellitus | 1.07 | 0.91-1.14 | 0.22 |
| Chronic Kidney Disease | 1.04 | 0.92-1.22 | 0.18 |
| Liver Disease | 1.12 | 1.01-1.25 | <0.01 |
| Alcohol Misuse | 0.91 | 0.77-1.17 | 0.48 |
| **Elixhauser Comorbidity Score (per 1 point)** | 1.02 | 1.01-1.04 | 0.01 |
| **No CPR Directive at ICU Admission** | 1.77 | 1.41-2.03 | <0.001 |
| **Location Prior to ICU Admission** |  |  |  |
| Hospital Wards | Ref |  |  |
| Emergency Department | 1.10 | 0.87-1.20 | 0.56 |
| Operating Room | 1.18 | 0.95-1.29 | 0.14 |
| Peripheral Hospital | 0.94 | 0.80-1.19 | 0.47 |
