# Supplementary material for: New-onset atrial fibrillation and associated outcomes and resource use among critically ill adults—a multicenter retrospective cohort study
Source: Crit Care. 2020 Jan 13;24:15. doi: 10.1186/s13054-020-2730-0 (PMC6958729; doi:10.1186/s13054-020-2730-0)
Supplement: Supplementary file 5 — Additional file 5 : Table S5. Multivariable Logistic Regression Model for hospital mortality among patients with new-onset atrial fibrillation (n = 1541). Multivariable Logistic Regression Model for hospital mortality among patients with new-onset atrial fibrillation (n = 1541). [file 13054_2020_2730_MOESM5_ESM.docx]

**Table S5**: Multivariable Logistic Regression Model for hospital mortality among patients with new-onset atrial fibrillation (*n* = 1,541). *Abbreviations:* MODS = Multiple Organ Dysfunction Score; ICU = Intensive Care Unit; CI = confidence interval; CPR = cardiopulmonary resuscitation

| **Variable** | **Odds Ratio** | **95% CI** | ***P Value*** |
| --- | --- | --- | --- |
| **Age (per 5 years)** | 1.05 | 1.02-1.11 | <0.001 |
| **Male Gender** | 0.98 | 0.90-1.10 | 0.33 |
| **MODS (per 1 point)** | 1.04 | 1.02-1.07 | <0.001 |
| **Comorbidities** |  |  |  |
| Congestive Heart Failure | 1.18 | 1.04-1.32 | <0.001 |
| Peripheral Vascular Disease | 1.08 | 0.89-1.29 | 0.23 |
| Hypertension | 0.96 | 0.86-1.08 | 0.51 |
| Chronic Obstructive Pulmonary Disease | 1.06 | 0.98-1.14 | 0.08 |
| Diabetes Mellitus | 1.04 | 0.94-1.15 | 0.19 |
| Chronic Kidney Disease | 1.05 | 0.95-1.18 | 0.20 |
| Liver Disease | 1.09 | 1.01-1.18 | 0.03 |
| Alcohol Misuse | 0.95 | 0.84-1.19 | 0.28 |
| **Elixhauser Comorbidity Score (per 1 point)** | 1.02 | 1.00-1.05 | 0.06 |
| **No CPR Directive at ICU Admission** | 1.44 | 1.17-1.80 | <0.001 |
| **Atrial Fibrillation Therapy** |  |  |  |
| Antiarrhythmic | Ref |  |  |
| Beta-blocker/Calcium Channel Blocker/Digoxin | 1.04 | 0.89-1.21 | 0.37 |
| Electrical Cardioversion | 1.06 | 0.79-1.38 | 0.81 |
| Combination | 0.94 | 0.81-1.09 | 0.22 |
| **Sustained Atrial Fibrillation** | 1.44 | 1.18-1.74 | <0.001 |
| **Location Prior to ICU Admission** |  |  |  |
| Emergency Department | Ref |  |  |
| Hospital Wards | 0.96 | 0.85-1.21 | 0.33 |
| Operating Room | 1.11 | 0.94-1.24 | 0.11 |
| Peripheral Hospital | 0.95 | 0.84-1.19 | 0.41 |
| **Most Responsible Diagnosis** |  |  |  |
| Other | Ref |  |  |
| Infection/Sepsis | 1.05 | 1.02-1.09 | 0.01 |
| Respiratory Failure | 1.28 | 1.09-1.47 | <0.001 |
| Trauma | 0.93 | 0.73-1.14 | 0.22 |
| Malignancy | 1.02 | 0.88-1.23 | 0.87 |
| Spontaneous Intracranial Hemorrhage | 2.01 | 1.68-2.40 | <0.001 |
| Stroke | 1.56 | 1.20-1.87 | <0.001 |
| Overdose/Poisoning | 0.42 | 0.35-0.72 | <0.001 |
| Renal Failure | 0.46 | 0.34-0.68 | <0.001 |
| Gastrointestinal Bleeding | 1.15 | 0.77-1.71 | 0.55 |
| Congestive Heart Failure | 1.14 | 0.87-1.59 | 0.72 |
| Cardiac Arrest | 1.88 | 1.53-2.30 | <0.001 |
| Seizures/Status Epilepticus | 0.66 | 0.59-0.98 | 0.03 |
| Diabetic Ketoacidosis | 0.67 | 0.35-0.94 | <0.01 |
