# Supplementary material for: New-onset atrial fibrillation and associated outcomes and resource use among critically ill adults—a multicenter retrospective cohort study
Source: Crit Care. 2020 Jan 13;24:15. doi: 10.1186/s13054-020-2730-0 (PMC6958729; doi:10.1186/s13054-020-2730-0)
Supplement: Supplementary file 6 — Additional file 6 : Table S6. Generalized Linear Model with gamma distribution and log link for total cost for entire study cohort (n = 15,014). Generalized Linear Model with gamma distribution and log link for total cost for entire study cohort (n = 15,014). [file 13054_2020_2730_MOESM6_ESM.docx]

**Table S6**: Generalized Linear Model with gamma distribution and log link for total cost for entire study cohort (*n* = 15,014). *Abbreviations:* MODS = Multiple Organ Dysfunction Score; ICU = Intensive Care Unit; CI = confidence interval; CPR = cardiopulmonary resuscitation

| **Variable** | **Cost Ratio** | **95% CI** | ***P Value*** |
| --- | --- | --- | --- |
| **Age (per 5 years)** | 0.94 | 0.89-1.01 | 0.07 |
| **Male Gender** | 1.02 | 0.89-1.11 | 0.14 |
| **New-Onset Atrial Fibrillation** | 1.09 | 1.02-1.21 | <0.01 |
| **MODS (per 1 point)** | 0.94 | 0.89-0.97 | <0.01 |
| **Comorbidities** |  |  |  |
| Congestive Heart Failure | 0.97 | 0.86-1.05 | 0.33 |
| Peripheral Vascular Disease | 1.03 | 0.95-1.11 | 0.27 |
| Hypertension | 1.01 | 0.91-1.09 | 0.63 |
| Chronic Obstructive Pulmonary Disease | 0.95 | 0.88-1.12 | 0.58 |
| Diabetes Mellitus | 1.03 | 0.94-1.10 | 0.41 |
| Chronic Kidney Disease | 0.96 | 0.83-1.18 | 0.39 |
| Liver Disease | 0.92 | 0.79-1.06 | 0.27 |
| Alcohol Misuse | 1.03 | 0.93-1.10 | 0.81 |
| **Elixhauser Comorbidity Score (per 1 point)** | 0.98 | 0.91-1.07 | 0.52 |
| **No CPR Directive at ICU Admission** | 0.82 | 0.70-0.93 | <0.001 |
| **Location Prior to ICU Admission** |  |  |  |
| Emergency Department | 0.94 | 0.88-1.07 | 0.48 |
| Hospital Wards | 1.06 | 0.89-1.22 | 0.53 |
| Operating Room | 1.10 | 0.98-1.26 | 0.07 |
| Peripheral Hospital | 0.91 | 0.80-1.04 | 0.10 |
| **Most Responsible Diagnosis** |  |  |  |
| Other | Ref |  |  |
| Infection/Sepsis | 1.07 | 0.90-1.19 | 0.42 |
| Respiratory Failure | 1.87 | 1.32-2.23 | <0.001 |
| Trauma | 1.47 | 1.13-1.77 | <0.001 |
| Malignancy | 0.92 | 0.80-1.08 | 0.35 |
| Spontaneous Intracranial Hemorrhage | 2.21 | 1.71-2.66 | <0.001 |
| Stroke | 1.13 | 1.00-1.26 | 0.05 |
| Overdose/Poisoning | 0.89 | 0.81-0.96 | <0.01 |
| Renal Failure | 0.83 | 0.65-0.94 | <0.001 |
| Gastrointestinal Bleeding | 1.10 | 0.98-1.19 | 0.09 |
| Congestive Heart Failure | 1.05 | 0.93-1.14 | 0.41 |
| Cardiac Arrest | 1.12 | 0.88-1.13 | 0.71 |
| Seizures/Status Epilepticus | 1.23 | 1.05-1.43 | <0.01 |
| Diabetic Ketoacidosis | 0.74 | 0.60-0.82 | <0.001 |
| **In-Hospital Death** | 0.62 | 0.57-0.70 | <0.001 |
| **Length of Stay (per 1 day)** | 1.03 | 1.02-1.05 | <0.001 |
| **Invasive Mechanical Ventilation** | 1.23 | 1.15-1.33 | <0.001 |
| **Renal Replacement Therapy** | 1.08 | 1.01-1.15 | <0.01 |
